# 112 Systematic Review of Validated Protocols for Fractional CO2 Laser Therapy in Burn Scar Revision

**DOI:** 10.1093/jbcr/iraf019.112

**Published:** 2025-04-01

**Authors:** Yossi Cohen, Andree-Anne Roy

**Affiliations:** University of Montreal; University of Montreal

## Abstract

**Introduction:**

Psychological, social, and functional well-being are impacted in individuals suffering from scars, particularly those affected by large area burns. Fractional CO2 (FCO2) laser therapy has emerged as a promising treatment for scar revision, offering improvements in appearance, texture and function. Despite growing in popularity, heterogeneous results are common, and a validated protocol for FCO2 laser therapy remains lacking, with current practices relying primarily on anecdotal physician experience. This review aims to identify validated protocols for the use of FCO2 laser therapy in burn patients.

**Methods:**

A literature review was conducted to amalgamate existing evidence on the use of FCO2 laser therapy for burn scar revision, with a focus on pinpointing validated treatment protocols for this patient population. PubMed was systematically searched using MeSH terms related to burn scar revision and FCO2 therapy, covering studies from 2000 onward. Studies were included if they explicitly addressed FCO2 laser therapy for the treatment of scars related to burn injuries. Animal studies, those not addressing FCO2 therapy for burn scars, studies conducted before the year 2000, and studies that focused on cosmetic use were excluded.

**Results:**

Fourteen studies were screened, with five undergoing full-text review. Of these, four studies met the inclusion criteria (Image 1). The studies provided insight into the application of FCO2 laser therapy for burn scar revision, but none offered a validated, evidence-based protocol. One study suggested a treatment algorithm based on expert opinion, while others emphasized the need for further research to determine optimal treatment parameters such as wavelength, laser strength, and treatment frequency.

**Conclusions:**

Conclusion: FCO2 laser therapy is a viable and effective treatment for improving scars in burn patients. However, the widespread discrepancy in treatment results and the absence of a standardized protocol necessitates further research to define optimal treatment parameters and combination therapies. Establishing a validated protocol is essential for maximizing clinical outcomes and patient satisfaction in burn victims.

**Applicability of Research to Practice:**

Deploying a validated and objective protocol for the use of FCO2 laser therapy is the future aim of the study. Despite its efficacy and promising results, the heterogeneity of FCO2 laser therapy is undeniable. Developing a standardized and objective protocol that can be validated may increase the effectiveness of FCO2 laser therapy, helping patients achieve improvements in the functional, social, and, most importantly, psychological arenas. However, ensuring that the gap does exist is the first step in addressing the void.

**Funding for the Study:**

N/A

